# From heart to kidney: FGF23, SuPAR, and NAG as early biomarkers of cardiorenal syndrome in acute heart failure

**DOI:** 10.1186/s12872-026-05777-x

**Published:** 2026-04-15

**Authors:** Islam M. El-Desouki, Nevin A. Salah, Hanaa M. M. Abdel Aziz, Omali Y. El‑khawaga

**Affiliations:** 1 Biochemistry Division, Chemistry Department, Faculty of Science, Mansoura university, Mansoura, 35516 Egypt; 2 Cardiovascular Medicine Department, Faculty of Medicine, Mansoura University, Mansoura, 35516 Egypt

**Keywords:** Acute heart failure, Cardiorenal syndrome, Kidney disease, fibroblast growth factor-23 (FGF23), soluble urokinase plasminogen activator receptor (SuPAR), N-acetyl-β-D-glucosaminidase (NAG)

## Abstract

**Background:**

Acute heart failure (AHF) often occurs concurrently with renal impairment, forming the clinical entity known as cardiorenal syndrome. Conventional biomarkers frequently do not identify early renal impairment in patients with AHF.

**Objectives:**

The present study aimed to assess the clinical applicability of fibroblast growth factor-23 (FGF23), soluble urokinase plasminogen activator receptor (SuPAR), and N-acetyl-β-D-glucosaminidase (NAG) as potential biomarkers for the discrimination of kidney disease in the setting of AHF.

**Patients and methods:**

This prospective observational pilot study was performed from 2024 to 2025 in the critical care units of Mansoura University. In total, 80 subjects were included and classified into four groups: healthy controls (*n* = 20), renal disease group (*n* = 20), AHF patients (*n* = 20), and patients with combined AHF and renal dysfunction (*n* = 20). Patients’ clinical, demographic, and laboratory data were recorded at the admission. Serum FGF23 and SuPAR were detected by ELISA, and NAG expression was analyzed by real-time quantitative PCR with internal control normalization. Statistical methods consisted of ANOVAs, Kruskal–Wallis analyses, chi-square tests, Spearman’s correlations, and receiver operating characteristic (ROC) curves, plus logistic regression with *p* < 0.05 indicative of statistical significance.

**Results:**

The AUC of FGF-23 and SuPAR to differentiate AHF from renal disease was 0.792, with an accuracy of 82.5%, greater than those of the parameters. FGF23 and SuPAR well distinguished RIF-AHF (AUC > 0.96). The NAG gene performed at 100% sensitivity and specificity with an AUC of 1.0 to distinguish AHF, RD, and MCR from controls. The association of NAG concentration with inflammatory markers, acid–base disturbances, hypoxia indices, and renal function parameters indicated early tubular injury. NAG detected coincident clinical strata with excellent sensitivity even though its specificity was much lower.

**Conclusions:**

FGF23, SuPAR, and NAG are strong discriminators of early-stage cardiorenal dysfunction in AHF. NAG gene expression, in particular, exhibits excellent diagnostic performance and mirrors important pathophysiological processes before conventional biomarkers. Admission, follow-up, and the response of these biomarkers to treatment could improve early diagnosis, risk stratification, and clinical management in patients with AHF and renal dysfunction.

## Introduction

Global heart failure (HF) is a major health issue. HF affects around 64 million people globally, causing significant morbidity and death [1]. The WHO estimates that 17.9 million people die yearly from cardiovascular diseases (CVDs), accounting for 31% of all fatalities. Exploratory diagnostic performance and precise risk stratification improve heart disease outcomes [2].

AHF is a common hospitalization and risk factor for renal disease. AHF patients commonly develop renal impairment or failure, prolonging hospital stays and increasing mortality [3]. Few pharmacological advancements have improved outcomes for AHF episodes, which are particularly sensitive to increasing morbidity and death [4]. Inability to recognize pathophysiologic processes during decompensation, failure to recognize optimal fluid status, and difficulty identifying patients with a worse prognosis and needing more aggressive interventions may explain this lack of improvement in AHF outcomes and care [5].

Some studies have shown up to 40% of severe decompensated heart failure patients had kidney disease [6]. The causes of kidney disease in AHF are complicated and multifaceted, with different pathways in different individuals. Therefore, exploratory diagnostic performance of acute cardiorenal syndrome improves prognosis and clinical results [7]. Serum creatinine is a poor indicator of renal disease due to its prolonged increase in circulation after the first renal insult [8]. This constraint has sparked research in new biomarkers that might identify kidney impairment earlier.

Among the emerging biomarkers, Fibroblast Growth Factor-23 (FGF23) has garnered significant attention due to its association with phosphate metabolism and cardiovascular risk [9]. FGF23 is mostly secreted by osteocytes and has recently been identified as crucial in regulating renal phosphate excretion, as well as being linked to negative cardiovascular outcomes, including heart failure, ventricular hypertrophy, and death [10]. Increased FGF23 levels are associated with myocardial remodelling and worse prognosis in heart failure and chronic kidney disease, suggesting that it is an integrative marker of early cardiorenal stress [10].

Another potentially attractive biomarker of chronic inflammation and immune activation is SuPAR [11]. As the soluble variant of the GPI-anchored uPAR, SuPAR is shed in response to immune cell activation and has been linked to the progression of kidney disease and cardiovascular events [12]. When added to traditional markers, including B-type natriuretic peptide (BNP), high SuPAR levels have been associated with incident heart failure, cardiovascular death, and hospitalization that might enhance risk discrimination in patients with or at risk for cardiorenal disease [11].

N-acetyl-β-D-glucosaminidase (NAG), a lysosomal enzyme located in renal proximal tubule cells, is an indicator of tubular damage and has been implicated as being involved in early kidney injury compared to filtration markers [13]. Increased urinary or serum NAG levels have been linked to kidney disease, progression of chronic kidney disease, and heart failure complications, which implies that measurement of these values is useful for identification of subclinical renal damage in the context of cardiorenal syndrome [14].

Although these biomarkers have clinical implications, their aggregating and comparing performance in detecting the early cardiorenal abnormality of AHF are commonly uninvestigated. The incorporation of FGF23, SuPAR, and NAG into the clinical evaluation may offer a more holistic picture of potential pathophysiological mechanisms, such as electrolyte and mineral metabolism (including sodium, potassium, and related metabolic parameters), inflammatory activation, and tubular damage, that would aid in earlier detection and intervention. The present study aimed to assess the clinical applicability of FGF23, SuPAR, and NAG as molecular and protein markers for the diagnosis of kidney disease in patients with AHF.

## Patients and methods

### Study design

Prospective observational pilot research was performed on patients with AHF admitted to the critical care units of Mansoura University Hospital, Egypt. The research was conducted at the Department of Cardiology at Mansoura University Hospital from 2024 to 2025. Following approval by the hospital’s ethics council, a total sample of 80 participants was enrolled, including 20 healthy individuals, 20 patients with chronic kidney disease, 20 patients with AHF without kidney disease, and 20 patients with combined AHF and acute kidney disease.

The research protocol received approval from the Ethical Committee of the Faculty of Medicine at Mansoura University (Institutional Review Board [IRB] code: MDP.24.05.153). All experimental methods, including specimen collection and biochemical analysis, were conducted in compliance with relevant ethical principles and legislation.

All participants underwent a clinical evaluation and, with the exception of the control group, received treatment from a physician, alongside the collection of demographic information (age, sex, weight, height, blood pressure, medication history, family history of kidney disease, and characteristics of the current illness). Standard laboratory evaluations were conducted, including serum creatinine, urine output, estimated glomerular filtration rate (GFR), urinary protein, and urinary albumin.

Patients with AHF were treated with guideline-directed medical therapy according to ESC guidelines: They received diuretics, vasodilators, inotropes, and renin–angiotensin system inhibitors when appropriate. Controls received no treatment.

### Study groups

The study population was divided into four groups based on clinical diagnosis and renal status.

- Control group: 20 healthy individuals without cardiac or renal diseases
- Chronic kidney disease (CKD)
- Acute heart failure (AHF) without evidence of kidney disease
- Combined acute heart failure and acute kidney injury (AHF with AKI)

### Clinical endpoints and definitions

The primary clinical endpoint of the present study was acute kidney disease in the setting of AHF, or type 1 cardiorenal syndrome. Acute Kidney disease, or acute kidney injury (AKI), was defined based on the following: Kidney Disease Improving Global Outcomes (KDIGO) criteria by an increase in serum creatinine of ≥ 0.3 mg/dL within 48 h from the baseline level at hospital admission, or a rise to > 1.5 times baseline over 7 days. The baseline creatinine was defined as the serum creatinine level measured at hospital admission, which was used as the reference value for subsequent comparisons and for applying the KDIGO criteria for the diagnosis of AKI. Urine output criteria were not utilized because of missing hourly documentation in a few patients. Secondary classification was according to the clinical diagnosis at admission, into: AHF no RFTwD, isolated RD, AHF and renal dysfunction, and healthy controls. Acute HF was defined according to ESC guidelines and required clinical signs of congestion supplemented by echocardiographic evidence and increased natriuretic peptides if available.

### Inclusion criteria & exclusion criteria

The study’s inclusion criteria encompass patients aged 18 to 65 years presenting with AHF and renal disease, irrespective of sex, including individuals with diabetes mellitus. The age limit of under 65 years was set to avoid mixing up the effects of being older and being weak, since these factors, along with other health issues, can separately influence biomarker levels and kidney function.

The exclusion criteria include individuals who have undergone transplantation, have malignancy, or suffer from lupus nephritis. Active cancer patients were excluded because of possible impacts on inflammatory and metabolic analytes. Lupus nephritis was not included to reduce potential confounding by ongoing immune-mediated renal inflammation, which could independently affect biomarker expression. Patients with a preceding solid organ transplant were excluded because of changed immunologic and renal physiology that may affect the interpretation of biomarkers.

### Sample collection

Five milliliters of peripheral blood was drawn. Two tubes were used for the gene expression analysis: one containing EDTA for total RNA extraction and the other separated serum for exosome miRNA separation using a gel tube. All blood and urine samples were collected within 24 h after hospital admission, before any meaningful therapeutic change was possible. The sampling time point for all study groups was standardized from a single-dose DF-DR treatment to limit variation due to treatment exposure.

## Methods

### Measurement of FGF23 level

FGF23 concentrations were measured employing a human fibroblast growth factor-23 ELISA reagent (catalog number E-EL-H1116, Elabscience^®^, Wuhan, China). The test exhibited a sensitivity of 6.1 pg/ml, with a detection range spanning from 15.63 to 1000 pg/ml, and no instances of cross-reactivity were reported.

### Measurement of serum SuPAR level

Serum SuPAR levels were measured utilizing the Elabscience^®^ Human Fibroblast Growth Factor 23 ELISA Kit (catalog number [E-EL-H2584]). The assay was conducted in accordance with the manufacturer’s protocol and exhibited high sensitivity and specificity for human SuPAR.

### NAG gene expression by quantitative real-time PCR

Whole blood was collected and the total RNA isolated with TRIzol™ Reagent (Cat. 15596026, Life Technologies, USA) following the manufacturer’s manual. In brief, blood samples were homogenized with TRIzol reagent, and phase separation was performed by the addition of chloroform. RNA in the aqueous phase was precipitated with isopropanol, washed with 75% ethanol, air-dried, and dissolved in RNase-free water. Concentration and purity of extracted RNA were spectrophotometrically determined, samples with an A260/A280 ratio higher than 1.8 being eligible for further analyses.

The cDNA was prepared from 1 µg of the total RNA with the QuantiTect Reverse Transcription Kit (Qiagen, USA). The protocol consisted of a genomic DNA removal step using gDNA Wipeout Buffer, followed by reverse transcription with random hexamer primers as per the manufacturer’s directions. The cDNA was kept at − 20 °C for real-time PCR (qPCR).

qRT-PCR Total RNA was submitted to reverse transcription, and the resulting cDNA was subjected to quantitative real-time PCR (Maxima SYBR Green/Fluorescein qPCR Master Mix) (Cat. K0243, Thermo Fisher Scientific, Waltham, MA, USA) by the Rotor-Gene Q real-time PCR system (Qiagen, USA). The primers were specific for the human N-acetyl-β-D-glucosaminidase (NAG) gene, and GAPDH was used as an internal control.

All assays were conducted in two replicates. NAG gene expression was normalized to β-actin expression; the relative NAG gene mRNA levels were determined by the comparative Ct method (2⁻ΔΔCt). The Cts of the NAG gene were normalised to the Cts of GAPDH (ΔCt), and fold changes were determined using data from control samples as reference [15] (Table 1).

**Table 1:** List of primer sequences

Genes	Sequence	Reference
NAGA Human qPCR Primer Pair (NM_000262)	TGCCTTATCGCTACCACTCCTC GAAGTTGGTTTCATCTCGGAGGC	OriGene
GAPDH Human qPCR Primer Pair (NM_002046)	GTCTCCTCTGACTTCAACAGCG ACCACCCTGTTGCTGTAGCCAA	

### Statistical analysis

The data was amended, encoded, and organized using IBM SPSS Statistics Version 25.0 and UNCTAD’s Handbook of Statistics. Descriptive statistics for both numerical and non-numerical data included mean, standard deviation, median, range, frequency, and percentage. The Shapiro-Wilk test confirmed normalcy. Analytical statistics included the Chi-Square test for categorical data, Fisher’s Exact test for low expected frequencies, One-Way ANOVA for parametric variable comparison, and Mann-Whitney and Kruskal-Wallis tests for non-parametric variables. Spearman’s rho was used for correlation analysis. ROC curve methodology was used to evaluate the diagnostic accuracy of individual biomarkers. Area under the curve (AUC) has been calculated with its 95% CIs. The optimal cut-off points were established based on an exploratory, data-driven approach with the Youden index. Sensitivities, specificities, positive predictive values (PPV), negative predictive values (NPV), and overall accuracies were estimated by their 95% CIs with the binomial Wilson method. The bootstrap resampling method was employed to perform internal validation for assessing model stability and compared to optimism bias. Univariate screening and multivariable models with adjusted ORs were used to construct logistic regression models. A *p*-value less than 0.05 was deemed significant.

## Results

### Baseline demographic and clinical characteristics of the study population by study group (Table 2)

The study cohorts (*n* = 20 per group) maintained a comparable age distribution (55.0–56.7 years), though the AHF with Kidney Disease group exhibited the highest prevalence of males (85%), smokers (85%), and a 100% frequency of all assessed comorbidities. Clinically, this combined group demonstrated the most severe physiological distress, characterized by significant hypoxemia (pO_2_ 36.0 ± 10.29 mmHg), marked elevation in liver enzymes (ALT: 212.3 ± 139.5 U/L), and the highest lipid profile (Total Cholesterol: 315.8 ± 16.28 mg/dL) compared to other groups. Furthermore, while isolated Kidney Disease patients presented with the highest creatinine (7.18 ± 0.78 mg/dL) and hyperkalemia (6.72 ± 0.14 mmol/L), the AHF with Kidney Disease cohort showed a distinct pattern of anemia (Hemoglobin: 9.37 ± 0.70 g/dL) and hypokalemia (2.88 ± 0.20 mmol/L), highlighting the complex biochemical shifts in cardiorenal syndrome (Table 2).

**Table 2:** A descriptive baseline characteristics of study population by study group

Variable	Control (*n* = 20)	Acute Heart Failure (*n* = 20)	Kidney Disease (*n* = 20)	AHF with Kidney Disease (*n* = 20)
Baseline demographics				
Age (years)	55.0 ± 9.20	56.10 ± 8.77	55.0 ± 8.76	56.70 ± 8.23
Male, n (%)	8 (40%)	12 (60%)	12 (60%)	17 (85%)
Smoking, n (%)	0 (0%)	12 (60%)	0 (0%)	17 (85%)
Comorbidities				
Hypertension, n (%)	0 (0%)	16 (80%)	20 (100%)	20 (100%)
Diabetes mellitus, n (%)	0 (0%)	12 (60%)	20 (100%)	20 (100%)
Coronary artery disease, n (%)	0 (0%)	20 (100%)	8 (40%)	20 (100%)
Valvular heart disease, n (%)	0 (0%)	12 (60%)	20 (100%)	20 (100%)
kidney disease, n (%)	0 (0%)	0 (0%)	20 (100%)	20 (100%)
Laboratory parameters:				
Hemoglobin (g/dL)	12.32 ± 0.62	12.24 ± 1.68	9.82 ± 0.80	9.37 ± 0.70
RBC (×10¹²/L)	4.13 ± 0.19	5.17 ± 0.82	3.83 ± 0.15	4.40 ± 0.14
WBC (×10⁹/L)	5.60 ± 1.24	7.48 ± 1.22	4.54 ± 0.14	11.32 ± 1.42
Platelets (×10⁹/L)	232.6 ± 41.59	265.0 ± 52.21	150.4 ± 11.23	244.8 ± 27.81
Sodium (mmol/L)	139.0 ± 2.68	153.8 ± 1.51	129.4 ± 2.48	142.6 ± 3.57
Potassium (mmol/L)	3.72 ± 0.08	3.28 ± 0.08	6.72 ± 0.14	2.88 ± 0.20
Creatinine (mg/dL)	0.82 ± 0.18	1.52 ± 0.12	7.18 ± 0.78	3.17 ± 0.35
ALT (U/L)	19.88 ± 2.11	28.18 ± 1.40	25.22 ± 1.13	212.3 ± 139.5
AST (U/L)	20.56 ± 2.71	42.06 ± 3.34	31.40 ± 1.01	112.9 ± 60.29
pH	7.41 ± 0.03	7.36 ± 0.04	7.31 ± 0.01	7.32 ± 0.10
pO₂ (mmHg)	93.6 ± 2.95	85.4 ± 5.45	70.0 ± 3.24	36.0 ± 10.29
pCO₂ (mmHg)	38.6 ± 3.08	38.2 ± 3.27	30.2 ± 1.64	30.25 ± 6.78
HCO₃ (mEq/L)	24.04 ± 0.57	21.40 ± 3.89	14.80 ± 1.10	17.39 ± 5.29
SO₂ (%)	98.6 ± 0.50	97.8 ± 1.01	95.0 ± 1.45	72.9 ± 11.87
Total cholesterol (mg/dL)	181.8 ± 17.76	256.4 ± 20.95	213.6 ± 22.03	315.8 ± 16.28
Triglycerides (mg/dL)	106.5 ± 20.50	169.7 ± 14.93	161.1 ± 13.61	199.7 ± 19.21
HDL (mg/dL)	45.35 ± 9.05	67.40 ± 8.93	57.55 ± 11.50	38.85 ± 5.50
LDL (mg/dL)	114.8 ± 11.41	155.1 ± 19.31	124.8 ± 27.36	233.7 ± 24.56
Troponin I (ng/mL)	0.10 ± 0.24	1.50 ± 0.50	0.06 ± 0.04	0.21 ± 0.05

*SD *Standard deviation, *Min* Minimum, *Max* Maximum, *U* Mann–Whitney test

* Significant when *p* value < 0.05

### Association between FGF23 levels and different parameters

Table 3 demonstrates the evaluation of FGF23 levels in relation to gender and smoking, revealing differing patterns across the studied groups. In cases of renal illness, males had markedly elevated levels of FGF23 compared to women (*p* < 0.001). Other groups did not show any statistically significant changes in FGF23 levels. The smoking status did not have a significant impact on FGF23 levels across all examined groups.

**Table 3:** Association between FGF-23 levels with gender and smoking

	FGF23 (pg/mL)			U	*p*
	Mean ± SD.	Median	Min. – Max.		
Gender					
Control					
Male, *n* = 8	160.1 ± 25.53	161.98	124.1–195.82	31.0	0.208
Female, *n* = 12	275.73 ± 177.67	219.23	110.55–667.4		
AHF					
Male, *n* = 12	323.49 ± 54.43	329.15	249.3–396.23	48.0	1.0
Female, *n* = 8	313.33 ± 114.08	320.28	194.41–433.12		
Kidney disease					
Male, *n* = 12	486.53 ± 46.48	467.51	421.7–552	0.011	< 0.001*
Female, *n* = 8	305.32 ± 34.92	297.35	276.36–385.8		
AHF and kidney disease					
Male, *n* = 13	628.14 ± 172.84	703.4	273.8–801.11	21.0	0.056
Female, *n* = 7	753.45 ± 48.14	769.66	650.3–792.5		
Smoking					
AHF					
Non-smoker, *n* = 8	313.3 ± 114.1	320.3	194.4–433.1	48.0	1.0
Smoker, *n* = 12	323.5 ± 54.43	329.2	249.3–396.2		
Kidney disease					
Non-smoker, *n* = 20	414.1 ± 99.98	446.7	276.4–552.0	-	-
AHF and kidney disease					
Non-smoker, *n* = 3	776.1 ± 5.66	778.5	769.7–780.2	9.0	0.093
Smoker, *n* = 17	653.6 ± 159.2	723.0	273.8 – 801.1		

*SD* Standard deviation, *Min* Minimum, *Max* Maximum, *U* Mann–Whitney test

*Significant when *p* value < 0.05

Table 4 displays the examination of FGF23 levels relative to comorbidities; hypertension, diabetes mellitus, and valvular heart disease did not seem to substantially influence FGF23 in the heart failure cohort. Furthermore, in the cohort with renal illness, CAD did not significantly influence FGF23 levels.

**Table 4:** Association between FGF23 levels and comorbidities

	FGF23 (pg/mL)			U	*p*
	Mean ± SD.	Median	Min. – Max.		
Hypertension					
AHF					
No, *n* = 4	418.9 ± 14.85	420.5	401.5–433.1	23.0	0.061
Yes, *n* = 16	294.6 ± 70.27	288.1	194.4–396.2		
DM					
AHF					
No, *n* = 8	313.3 ± 114.1	320.3	194.4–433.1	48.0	1.0
Yes, *n* = 12	323.5 ± 54.43	329.2	249.3–396.2		
CAD					
Kidney disease					
No, *n* = 12	413.3 ± 69.14	446.7	300.2–477.8	48.0	1.0
Yes, *n* = 8	415.2 ± 140.1	415.6	276.4–552.0		
VHD					
AHF					
No, *n* = 8	320.7 ± 65.98	320.0	249.3–396.2	48.0	1.0
Yes, *n* = 12	318.6 ± 92.29	329.2	194.4–433.1		

*SD* Standard deviation, *Min* Minimum, *Max*Maximum, *U* Mann–Whitney test

*Significant when *p* value < 0.05

Table 5 shows the relationships between FGF23 levels and several clinical and biochemical parameters across all examined groups. In healthy controls, FGF23 had positive connections with platelet count and serum potassium while showing substantial unfavorable interactions with pO₂ and pCO₂. In AHF, FGF23 exhibited a substantial negative association with age, white blood cells, alanine aminotransferase, pH, partial pressure of carbon dioxide, and bicarbonate, while showing a positive correlation with partial pressure of oxygen.

Patients with renal illness demonstrated favorable associations of FGF23 with hemoglobin, red blood cell count, potassium, and oxygenation indices, with negative correlations with white blood cells, creatinine, and liver enzymes. In individuals with simultaneous acute cardiac failure and renal dysfunction, FGF23 has shown substantial relationships with red blood cells, sodium, potassium, pH, and oxygen saturation.

**Table 5:** Correlation between FGF23 levels and different parameters

	FGF23							
	Control *N* = 20		AHF *n* = 20		Kidney disease *n* = 20		AHF and kidney disease *n* = 20	
	*p*		*p*		*p*		*p*	*p*
Age	0.127	0.593	-0.456	0.043*	0.005	0.985	-0.211	0.371
Hb	-0.233	0.323	0.098	0.681	0.601*	0.005*	0.234	0.321
RBCs	0.066	0.782	0.392	0.087	0.601*	0.005*	0.449*	0.047*
WBC	0.141	0.553	-0.883*	< 0.001*	-0.796*	< 0.001*	-0.074	0.757
Platelet	0.506*	0.023*	0.294	0.208	0.300	0.198	-0.354	0.125
Na	0.233	0.323	0.101	0.673	0.012	0.959	0.525*	0.017*
K	0.637*	0.003*	-0.207	0.382	0.840*	< 0.001*	0.615*	0.004*
Creatinine	-0.080	0.738	0.101	0.673	-0.674*	0.001*	-0.181	0.446
ALT	0.184	0.438	-0.491*	0.028*	-0.570*	0.009*	0.318	0.172
AST	0.184	0.438	0.098	0.681	0.282	0.228	0.031	0.896
pH	0.233	0.323	-0.956*	< 0.001*	-0.277	0.237	0.652*	0.002*
pO₂	-0.909*	< 0.001*	0.687*	0.001*	0.871*	< 0.001*	-0.321	0.168
pCO₂	-0.717*	< 0.001*	-0.785*	< 0.001*	-0.129	0.588	0.440	0.052
HCO₃	0.055	0.817	-0.883*	< 0.001*	-0.129	0.588	0.236	0.317
SO₂	0.088	0.711	-0.439	0.053	0.871*	< 0.001*	0.565*	0.009*
TC	0.367	0.111	-0.103	0.665	-0.360	0.119	-0.012	0.960
TG	-0.141	0.553	-0.248	0.292	0.316	0.174	-0.345	0.137
HDL	0.095	0.689	-0.301	0.198	-0.305	0.191	0.161	0.496
LDL	0.407	0.075	0.188	0.427	-0.287	0.219	0.023	0.922
Troponin I	0.002	0.992	-0.217	0.357	0.084	0.725	-0.367	0.112

ρ, Spearman’s rho correlation coefficient

*Significant when *p* value < 0.05

### Association between SuPAR levels with different parameters

Table 6 demonstrates that SuPAR levels varied according to smoking status within the AHF group. Smoking is associated with markedly increased SuPAR levels in AHF. No substantial changes in SuPAR levels were observed depending on sex or smoking status among the examined groups.

**Table 6:** Association between SuPAR levels with gender and smoking among all studied groups

	SuPAR (pg/mL)			U	*p*
	Mean ± SD.	Median	Min. – Max.		
Gender					
Control					
Male, *n* = 8	3.73 ± 1.79	3.01	2.16–6.85	78.0	0.091
Female, *n* = 12	1.38 ± 0.42	1.35	0.91–2		
AHF					
Male, *n* = 12	2.94 ± 0.95	2.51	2.14–4.81	12.0	0.104
Female, *n* = 8	2.12 ± 0.28	2.17	1.72–2.39		
Kidney disease					
Male, *n* = 12	4.72 ± 1.2	5.26	2.24–5.71	32.0	0.238
Female, *n* = 8	5.21 ± 1.05	5.25	4.1–6.37		
AHF and kidney disease					
Male, *n* = 13	6.77 ± 1.67	7.18	3.33–8.71	37.0	0.536
Female, *n* = 7	7.4 ± 0.72	7.4	6.2–8.5		
Smoking					
AHF					
Non-smoker, *n* = 8	2.12 ± 0.28	2.17	1.72–2.39	84.0*	0.004*
Smoker, *n* = 12	2.94 ± 0.95	2.51	2.14–4.81		
Kidney disease					
Non-smoker, *n* = 20	4.91 ± 1.14	5.26	2.24–6.37	-	-
AHF and kidney disease					
Non-smoker, *n* = 3	7.61 ± 0.28	7.50	7.40–7.93	18.0	0.479
Smoker, *n* = 17	6.88 ± 1.52	7.18	3.33–8.71		

*SD* Standard deviation, *Min* Minimum, *Max* Maximum, *U* Mann–Whitney test.

*Significant when *p* value < 0.05

Table 7 illustrates the influence of comorbidities on SuPAR levels, demonstrating that AHF patients with hypertension or diabetes had markedly increased values. Neither CAD nor VHD influenced SuPAR levels in the renal disease or AHF cohorts.

**Table 7:** Association between SuPAR levels with comorbidities

	SuPAR (pg/mL)			U	*p*
	Mean ± SD.	Median	Min. – Max.		
Hypertension					
AHF					
No, *n* = 4	1.87 ± 0.12	1.88	1.72–2.0	64.0*	< 0.001*
Yes, *n* = 16	2.80 ± 0.85	2.43	2.14–4.81		
DM					
AHF					
No, *n* = 8	2.12 ± 0.28	2.17	1.72–2.39	84.0*	0.004*
Yes, *n* = 12	2.94 ± 0.95	2.51	2.14–4.81		
CAD					
Kidney disease					
No, *n* = 12	5.71 ± 0.40	5.66	5.11–6.37	20.0	0.073
Yes, *n* = 8	3.72 ± 0.75	4.05	2.24–4.50		
VHD					
AHF					
No, *n* = 8	3.16 ± 1.12	2.73	2.14–4.81	27.0	0.115
Yes, *n* = 12	2.25 ± 0.31	2.38	1.72–2.66		

*SD* Standard deviation, *Min* Minimum, *Max* Maximum, *U* Mann–Whitney test

*Significant when *p* value < 0.05

The Table 8 depicts the relationships between SuPAR levels and several clinical and biochemical parameters across distinct populations. In healthy controls, SuPAR demonstrated substantial positive relationships with hemoglobin, red blood cell count, creatinine levels, oxygenation indices, and troponin I. In individuals with AHF, SuPAR had a positive association with red blood cells, platelets, electrolytes, creatinine, liver enzymes, and oxygen saturation, while exhibiting a substantial negative correlation with pO₂.

In renal disease, hemoglobin and red blood cell counts exhibit negative correlations, while creatinine and white blood cell counts demonstrate robust positive associations. In instances of simultaneous AHF and renal dysfunction, SuPAR showed strong relationships with red blood cells, white blood cells, and sodium levels, while exhibiting negative correlations with platelet counts and liver transaminases.

**Table 8:** Correlation between SuPAR levels with different parameters

	SuPAR							
	Control *N* = 20		AHF *n* = 20		Kidney disease *n* = 20		AHF and kidney disease *n* = 20	
		*p*		*p*		*p*		*p*
Age	-0.170	0.475	-0.139	0.559	0.229	0.331	-0.017	0.942
Hb	0.846*	< 0.001*	0.362	0.117	-0.491*	0.028*	0.150	0.529
RBCs	0.679*	0.001*	0.730*	< 0.001*	-0.491*	0.028*	0.630*	0.003*
WBC	0.380	0.098	0.037	0.878	0.654*	0.002*	0.667*	0.001*
Platelet	-0.220	0.351	0.626*	0.003*	0.294	0.208	-0.455*	0.044*
Na	-0.110	0.643	0.258	0.272	0.098	0.681	0.566*	0.009*
K	-0.084	0.725	0.702*	0.001*	0.101	0.673	-0.152	0.523
Creatinine	0.454*	0.044*	0.674*	0.001*	0.687*	0.001*	0.285	0.223
ALT	0.110	0.643	0.620*	0.004*	0.392	0.087	-0.766*	< 0.001*
AST	0.110	0.643	0.712*	< 0.001*	-0.687*	0.001*	-0.864*	< 0.001*
pH	-0.110	0.643	0.135	0.569	0.151	0.525	-0.075	0.754
pO₂	0.616*	0.004*	-0.448*	0.048*	-0.098	0.681	-0.301	0.198
pCO₂	0.527*	0.017*	0.141	0.553	0.201	0.395	0.218	0.355
HCO₃	0.221	0.350	0.184	0.437	0.201	0.395	0.143	0.546
SO₂	0.602*	0.005*	0.645*	0.002*	-0.098	0.681	-0.226	0.338
TC	-0.238	0.313	0.133	0.575	0.216	0.361	0.079	0.742
TG	0.331	0.154	0.025	0.918	-0.042	0.860	0.184	0.438
HDL	-0.239	0.311	0.110	0.644	0.140	0.556	0.242	0.305
LDL	-0.180	0.449	0.135	0.571	0.067	0.779	0.047	0.843
Troponin I	0.452*	0.045*	0.204	0.387	-0.363	0.116	-0.216	0.361

*ρ*, Spearman’s rho correlation coefficient

*Significant when *p* value < 0.05

### Association between NAG gene expression and different parameters

Table 9 demonstrates that NAG gene expression showed no significant differences related to gender or smoking status among the studied groups.

**Table 9:** Association between NAG gene expression with gender and smoking

	NAG gene expression			U	*p*
	Mean ± SD.	Median	Min. – Max.		
Gender					
AHF					
Male, *n* = 12	1.46 ± 0.07	1.50	1.30–1.50	44.0	0.792
Female, *n* = 8	1.51 ± 0.22	1.50	1.30–1.90		
Kidney disease					
Male, *n* = 12	1.71 ± 0.12	1.70	1.60–1.90	48.0	1.0
Female, *n* = 8	1.78 ± 0.30	1.75	1.50–2.10		
AHF and kidney disease					
Male, *n* = 17	2.08 ± 0.30	2.00	1.50–2.50	44.5	0.938
Female, *n* = 3	2.17 ± 0.06	2.20	2.10–2.20		
Smoking					
AHF					
Non-smoker, *n* = 8	1.51 ± 0.22	1.50	1.30–1.90	44.0	0.792
Smoker, *n* = 12	1.46 ± 0.07	1.50	1.30–1.50		
Kidney disease					
Non-smoker, *n* = 20	1.74 ± 0.20	1.70	1.50–2.10	-	-
AHF and kidney disease					
Non-smoker, *n* = 3	2.17 ± 0.06	2.20	2.10–2.20	21.50	0.689
Smoker, *n* = 17	2.08 ± 0.30	2.00	1.50–2.50		

*SD* Standard deviation, *Min* Minimum, *Max* Maximum, *U* Mann–Whitney test

*Significant when *p* value < 0.05

The data demonstrates the impact of comorbidities on NAG gene expression, revealing that in AHF, patients with hypertension exhibited significantly increased expression compared to those without. Conversely, NAG gene expression was unaffected by comorbidities in other examined groups. (Table 10).

**Table 10:** Association between NAG gene expression and comorbidities

	NAG gene expression			U	*p*
	Mean ± SD.	Median	Min. – Max.		
Hypertension					
AHF					
No, *n* = 4	1.33 ± 0.05	1.30	1.30–1.40	60.0*	0.005*
Yes, *n* = 16	1.52 ± 0.14	1.50	1.30–1.90		
DM					
AHF					
No, *n* = 8	1.51 ± 0.22	1.50	1.30–1.90	44.0	0.792
Yes, *n* = 12	1.46 ± 0.07	1.50	1.30–1.50		
CAD					
Kidney disease					
No, *n* = 12	1.83 ± 0.20	1.85	1.60–2.10	14.0	0.067
Yes, *n* = 8	1.59 ± 0.10	1.55	1.50–1.70		
VHD					
AHF					
No, *n* = 8	1.50 ± 0.0	1.50	1.50–1.50	32.0	0.238
Yes, *n* = 12	1.47 ± 0.19	1.40	1.30–1.90		

*SD* Standard deviation, *Min* Minimum, *Max* Maximum, *U* Mann–Whitney test

*Significant when *p* value < 0.05

Table 11 illustrates the relationships between NAG gene expression and several clinical and biochemical indicators among the studied groups. In AHF, NAG expression exhibited significant positive correlations with white blood cells, pH, pCO₂, and HCO₃, while demonstrating an inverse relationship with oxygen tension.

**Table 11:** Correlation between NAG gene expression and different parameters

	NAG gene expression					
	AHF *n* = 20		Kidney disease *n* = 20		AHF and kidney disease *n* = 20	
		*p*		*p*		*p*
Age	0.287	0.220	0.023	0.922	-0.005	0.985
Hb	-0.434	0.056	-0.012	0.958	-0.200	0.398
RBCs	-0.256	0.277	-0.012	0.958	-0.108	0.650
WBC	0.613*	0.004*	0.472*	0.035*	-0.034	0.888
Platelet	-0.434	0.056	0.871*	< 0.001*	0.576*	0.008*
Na	0.406	0.075	0.759*	< 0.001*	0.139	0.558
K	0.067	0.778	0.064	0.789	0.397	0.083
Creatinine	0.118	0.620	0.610*	0.004*	-0.539*	0.014*
ALT	0.256	0.277	0.684*	0.001*	0.566*	0.009*
AST	-0.128	0.591	-0.622*	0.003*	0.314	0.177
pH	0.878*	< 0.001*	0.696*	0.001*	0.345	0.136
pO₂	-0.946*	< 0.001*	0.187	0.431	0.297	0.204
pCO₂	0.792*	< 0.001*	0.842*	< 0.001*	0.218	0.357
HCO₃	0.946*	< 0.001*	0.842*	< 0.001*	-0.625*	0.003*
SO₂	0.114	0.631	0.187	0.431	0.441	0.051
TC	-0.108	0.651	0.143	0.548	0.037	0.877
TG	-0.057	0.812	0.288	0.218	-0.127	0.594
HDL	0.125	0.600	0.060	0.802	0.094	0.693
LDL	-0.172	0.468	-0.113	0.635	0.052	0.827
Troponin I	0.116	0.626	0.041	0.862	0.037	0.878

*ρ*, Spearman’s rho correlation coefficient

*Significant when *p* value < 0.05

The NAG expression in renal disease had a substantial link with WBCs, platelets, salt, creatinine, ALT, pH, pCO₂, and HCO₃, while demonstrating a significant negative correlation with AST. The NAG expression exhibited a positive correlation with platelet count and ALT, while demonstrating an inverse relationship with creatinine and bicarbonate.

### Correlation between novel biomarkers among different studied groups

**Fig. 1:**
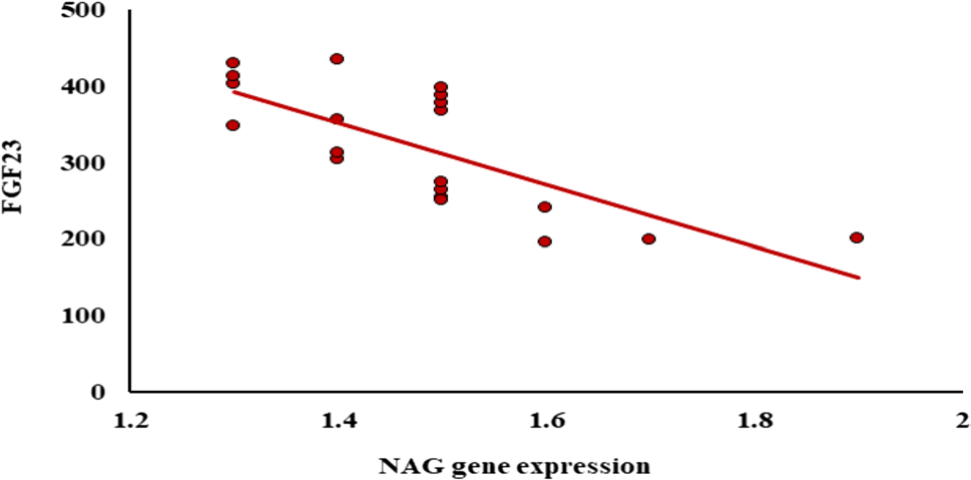
Correlation between FGF23 and NAG gene expression among AHF

Among all examined groups, the expressions of FGF23, SuPAR, and NAG genes demonstrated significant positive correlations with one another. As illustrated in Fig. 1.

**Fig. 2:**
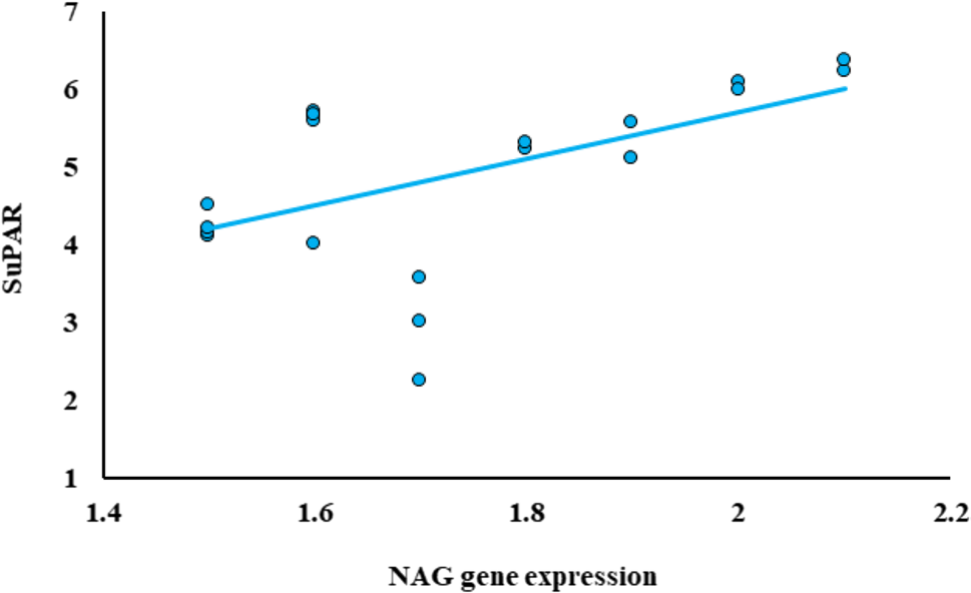
Correlation between SuPAR and NAG gene expression among kidney disease

Figure 2 shows substantial connections among the new biomarkers within the AHF population. The expression of the NAG gene has a substantial negative correlation with FGF23.

**Fig. 3:**
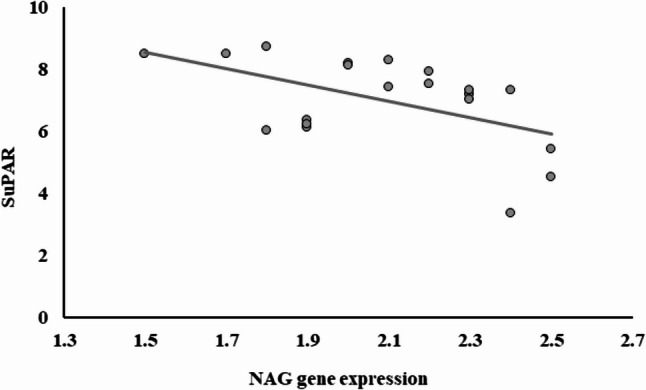
Correlation between SuPAR and NAG gene expression among AHF and kidney disease

Figure 3 clarifies the relationships among the new biomarkers within the renal disease cohort. SuPAR has substantial positive associations with NAG.

**Fig. 4:**
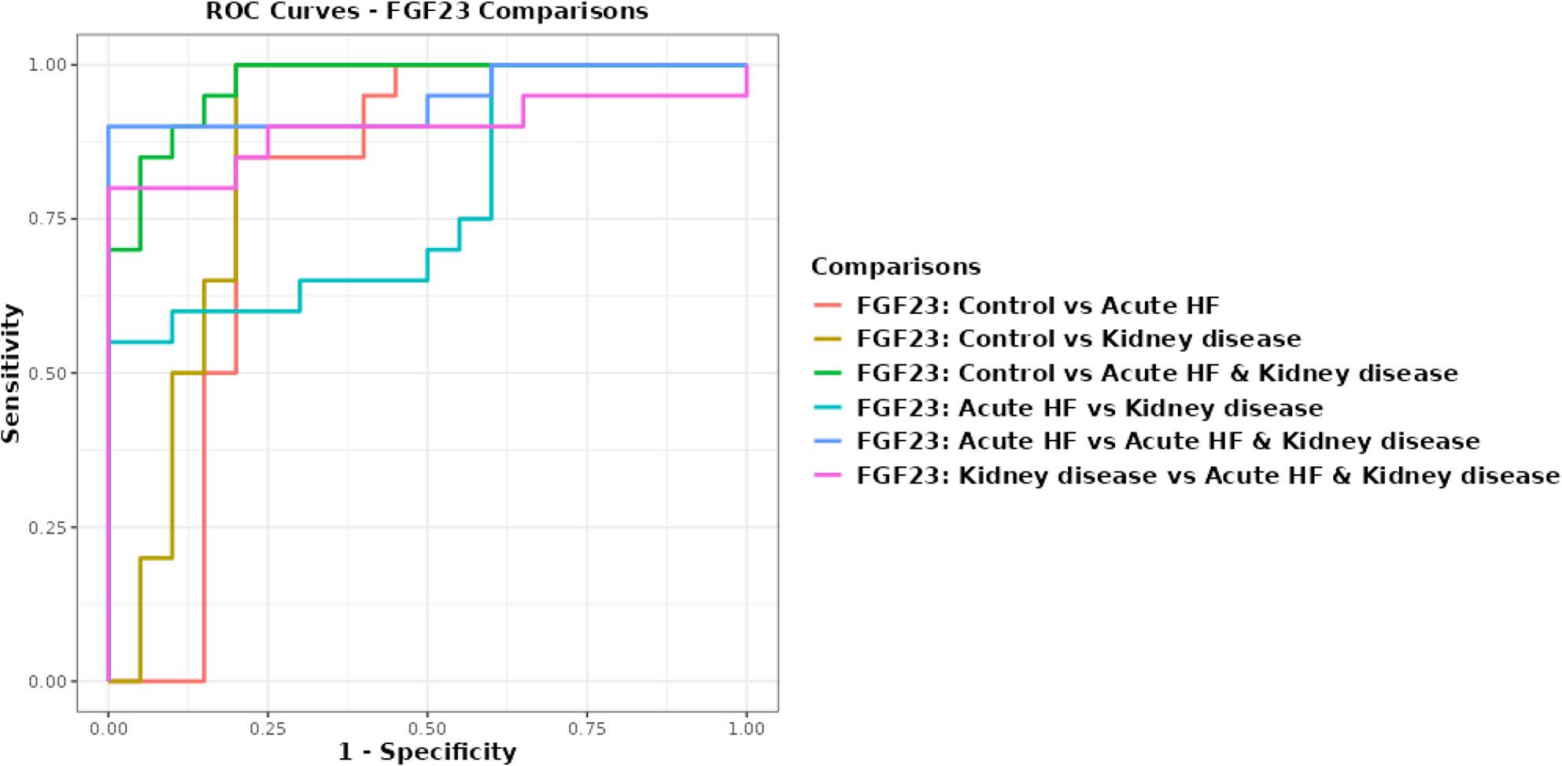
ROC Curve for FGF23 biomarkers for discrimination between AHF & kidney disease and control group

Figure 4 illustrates the association results for individuals with simultaneous AHF and renal illness. Significant negative associations existed between SuPAR and NAG.

### Validity of novel biomarkers for discrimination between the studied groups

FGF23 had a greater exploratory diagnostic effectiveness (AUC = 0.792 (95% CI = 0.632–0.953), 82.5% (95% CI = 68.1–91.3) accuracy) than SuPAR (AUC = 0.712 (95% CI = 0.540–0.885), 72.5% (95% CI = 57.2–83.9) accuracy). FGF23, SuPAR, and NAG showed good discriminatory ability in distinguishing renal illness patients from healthy controls. FGF23 and SuPAR showed AUCs of 0.970 (95% CI = 0.927-1.0) and 0.968, (95% CI = 0.922-1.0) respectively, for distinguishing AHF with kidney disease from controls, with an accuracy of 90.0 (95% CI = 76.9–96.0) for both; FGF23 offers clinically meaningful discrimination (AUC = 0.868 (0.738–0.997)). This method has been shown to be effective in distinguishing patients with renal illness from healthy controls. These findings are interpreted as exploratory diagnostic discrimination rather than definitive validation.

**Fig. 5:**
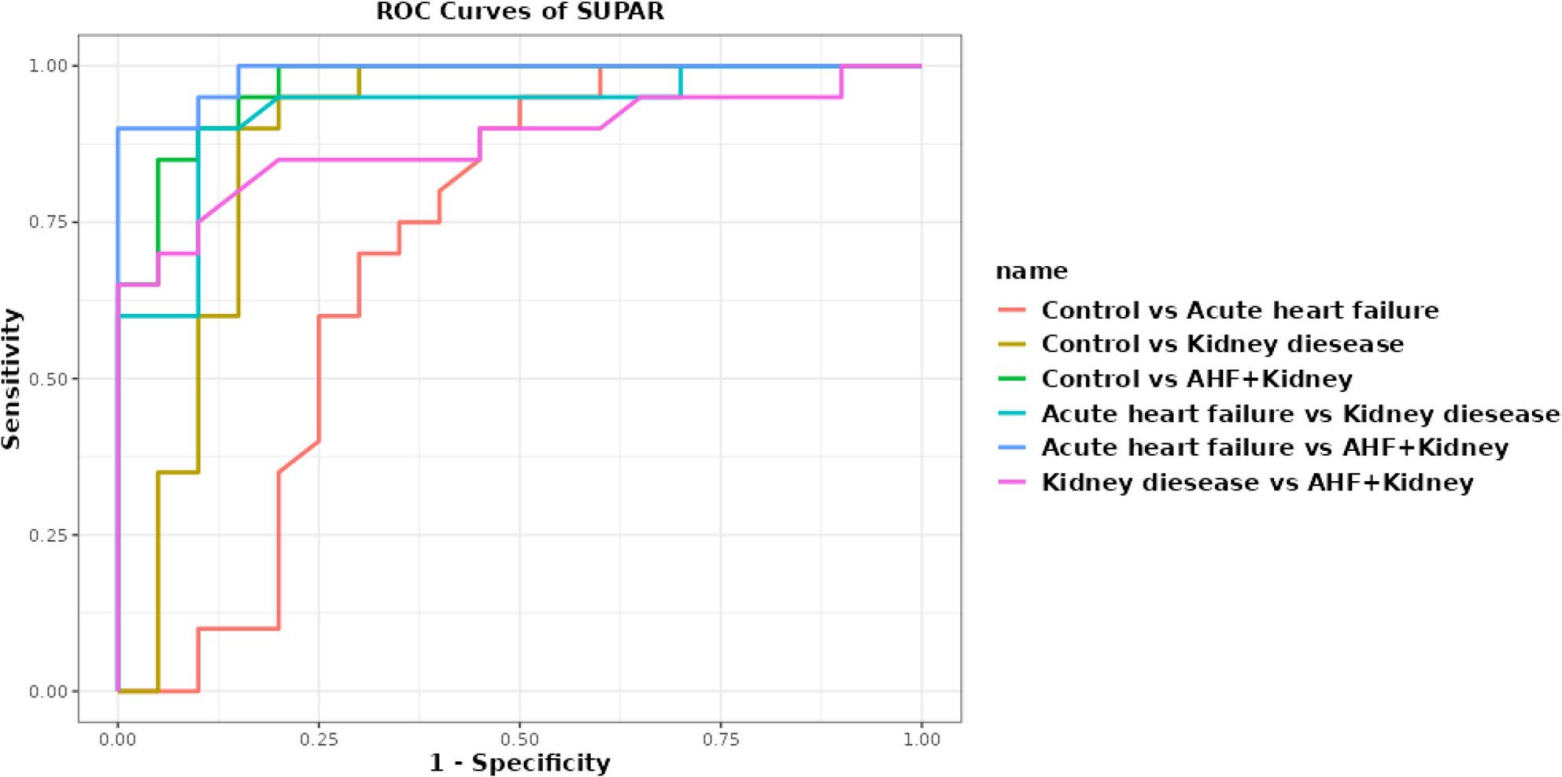
ROC Curve for SUPAR biomarkers for discrimination between AHF & kidney disease and control group

The AUC for FGF23 was 0.895 (95% CI = 0.779-1.0) (accuracy of 90% (95% CI = 80.7–99.3)) in distinguishing AHF with renal problems from kidney disease. FGF23 demonstrated high discriminatory performance in distinguishing AHF with renal impairment from AHF alone (AUC > 0.94, 95% accuracy). As illustrated in Figs. 4 and 5.

SuPAR demonstrated strong discriminatory capacity of AHF, both with and without renal impairment (AUC = 0.988 (95% CI = 0.964-1.0), accuracy 95% (95% CI = 83.5–98.6)). The discriminating capability of SuPAR was substantial (AUC = 0.879 (95% CI = 0.764–0.993), accuracy: 82.5% (95% CI = 70.7–94.3)). It has shown substantial diagnostic effectiveness in distinguishing patients with renal illness from healthy controls. This is depicted in Fig. 5.

**Fig. 6:**
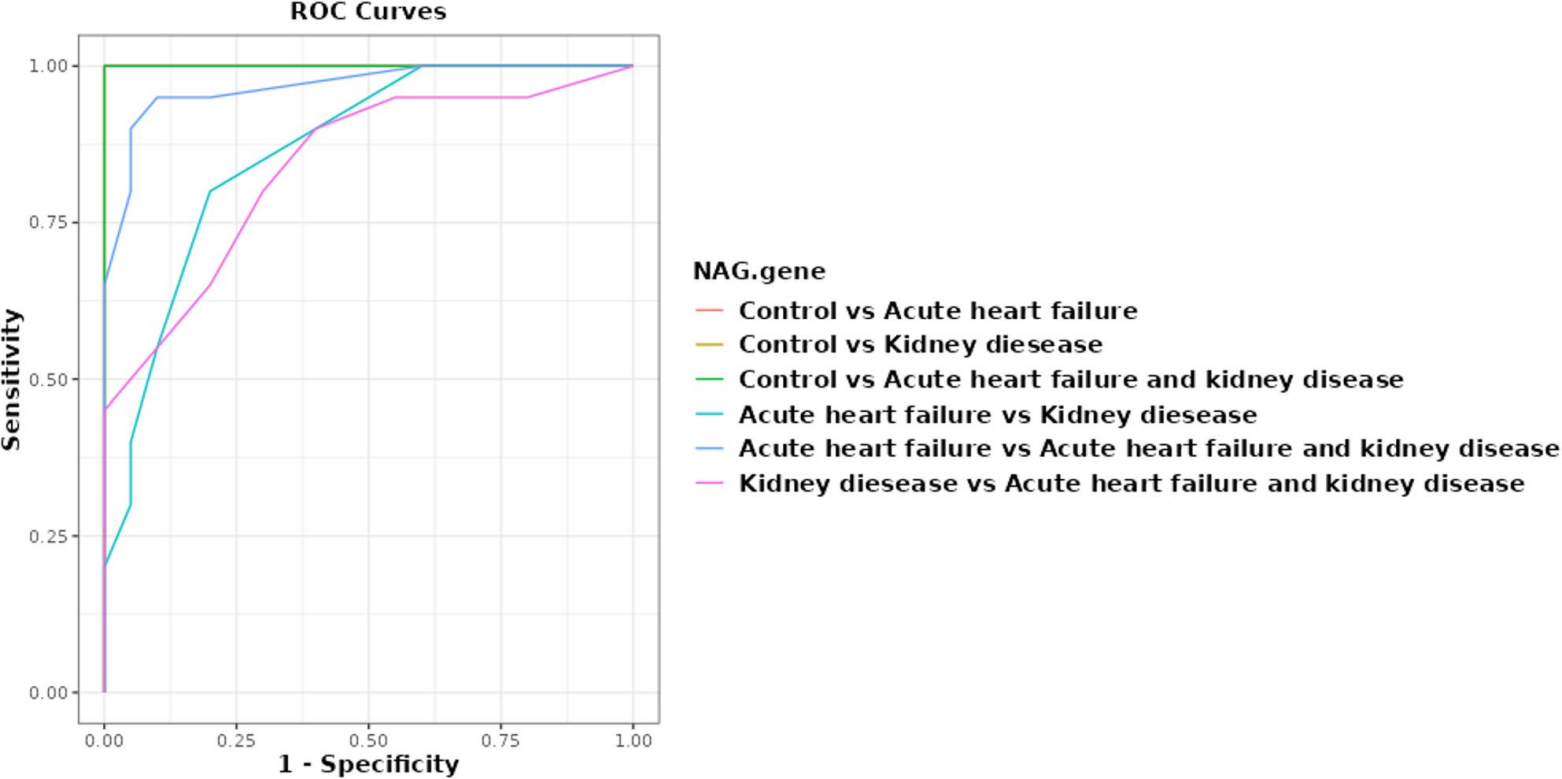
ROC Curve for NAG biomarkers for discrimination between AHF & kidney disease and control group

The ROC curve assessed the markers’ ability to distinguish patients from controls. The gene expression biomarker NAG had very high discriminatory performance in distinguishing AHF from control (AUC = 1.0) and sensitivity, specificity, PPV, NPV, and accuracy of 100%. NAG had perfect accuracy (AUC = 1.0, sensitivity 100% (95% CI = 83.9–100), specificity 100% (95% CI = 83.9–100)). Given the pilot sample size, these results are interpreted as exploratory. NAG also demonstrated strong discriminatory ability for AHF associated with renal impairment in control groups, with an AUC of 1.0, high sensitivity, specificity, PPV, NPV, and accuracy. NAG, the gene expression biomarker, distinguished AHF with renal impairment from acute heart failure alone with 92.5% (95% CI = 80.1–97.4) accuracy and 95% (95% CI = 76.4–99.1) sensitivity. The biomarker distinguished AHF with kidney disease from renal disease alone. The gene expression marker had moderate to high performance (AUC: 0.839 (95% CI = 0.715–0.963)), sensitivity 90.0 (95% CI = 76.8–100.0) and specificity of 60.0 (95% CI = 38.5–81.5) as shown in Fig. 6.

Overall, the biomarker analyses are presented as exploratory diagnostic discrimination in a pilot cohort and not as definitive validation.

### Exploratory diagnostic performance of susceptibility to AHF with kidney disease

We performed a multivariable logistic regression analysis to investigate factors related to susceptibility of AHF with KDD. In all models, a similar pattern was observed with circulating biomarkers (FGF23 and SuPAR) and NAG gene expression marker independently associated with the combined cardiorenal phenotype. The results are consistent with a multimodal risk profile whereby metabolic factors, circulating biomarkers, and gene expression markers collectively define predisposition to AHF-Renal (Table 12).

**Table 12:** Logistic regression analysis as an exploratory diagnostic tool for AHF with kidney disease susceptibility

	on top of healthy subjects							on top of AHF						on top of kidney disease				
	Univariate				Multivariate			Univariate			Multivariate			Univariate			Multivariate	
	*P*	OR	95% CI	*P*	OR	95% CI	*P*	OR	95% CI	*P*	OR	95% CI	*P*	OR	95% CI	*P*	OR	95% CI
Gender	0.114	0.528	0.239–1.166				0.744	0.875	0.392–1.953				0.744	0.875	0.392–1.953			
Age	0.522	1.013	0.974–1.053				0.416	0.986	0.952–1.021				0.849	1.003	0.968–1.040			
TC	< 0.001*	1.010	1.009–1.011	0.002*	1.005	1.002–1.008	0.107	1.177	0.965–1.435				0.398	1.091	0.892–1.333			
TG	< 0.001*	1.013	1.011–1.015	0.262	1.011	0.995–1.022	0.002*	1.073	1.027–1.121	0.315	1.121	0.979–1.272	0.051	1.225	1.001–1.502			
HDL	0.014*	0.919	0.859–0.983	0.002*	0.992	0.987–0.997	0.063	0.769	0.582–1.015				0.005*	0.800	0.686–0.935	0.255	0.993	0.989–1.008
LDL	< 0.001*	1.011	1.010–1.012	0.231	1.098	0.995–2.021	0.001*	1.058	1.024–1.094	0.004*	1.061	1.023–1.122	< 0.001*	1.037	1.018–1.056	0.058	1.004	1.002–1.009
Troponin I	0.069	6.404	0.866–7.336				0.003*	0.695	0.545–0.885	0.411	0.946	0.906–1.287	< 0.001*	4.157	3.181–5.432	0.036*	1.161	1.010–1.335
FGF23	< 0.001*	1.006	1.003–1.009	0.028*	1.453	1.287–3.043	0.001*	1.009	1.004–1.014	< 0.001*	1.198	1.044–1.214	< 0.001*	1.007	1.003–1.010	< 0.001*	1.125	1.103–1.309
SuPAR	< 0.001*	1.948	1.402–2.707	0.024*	1.009	1.988–1.030	0.001*	2.834	1.510–5.317	< 0.001*	1.058	1.038–1.079	< 0.001*	1.988	1.368–2.888	< 0.001*	1.026	1.018–1.034
NAG gene expression	< 0.001*	3.180	2.770–3.650	0.005*	1.200	1.056–1.363	< 0.001*	1.910	1.336–2.730	0.001*	1.143	1.053–1.241	0.001*	1.407	1.160–1.706	0.003*	1.044	1.015–1.074

*OR* Odd Ratio, *CI* Confidence interval

*Significant when *p* value < 0.05

## Discussion

This research assessed the correlation between renal function and the prognostic efficacy of FGF23, SuPAR, and NAG in patients with AHF. Acute cardiorenal syndrome results from sudden cardiac decline causing renal dysfunction, mostly due to renal venous congestion and increased neurohumoral activity, including stimulation of the sympathetic nervous system and the renin–angiotensin–aldosterone system [16]. Declining renal function affects 11–40% of AHF patients and correlates with extended hospitalization and increased death rates [17,18]. While serum creatinine serves as the conventional indicator for kidney disease, its delayed elevation and vulnerability to extrarenal influences constrain its dependability in acute circumstances [19]. This highlights the need for early indicators that are related to pathophysiology, which may help doctors to quickly identify patients at high risk for both heart and kidney problems for the best AHF management and prognosis.

Vascular dysfunction, abnormal electrolyte and mineral metabolism (including sodium, potassium, and related metabolic parameters), and left ventricular hypertrophy have all been mechanistically connected to FGF23, a phosphaturia hormone that is raised early in chronic kidney disease [20–23]. Our results indicate that in instances of renal disease, males exhibited significantly higher levels of FGF23 than females (*p* < 0.001). This is in agreement with other studies [23–25]. Of these, FGF23 showed fair diagnostic efficacy for isolated renal disease versus controls (AUC = 0.792; accuracy, 82.5%) and excellent performance in differentiating combined AHF with renal impairment from controls (AUC = 0.970) and from isolated renal disease (AUC = 0.895) in our cohort. This result is consistent with previous studies [26–28]. This finding is supported by clinical observation that elevated FGF23 levels are associated with cardiovascular events and heart failure outcomes in chronic kidney disease and general populations, raising its prominence as a cardiorenal risk factor.

Interestingly, controversy should also be noted in the literature on independent association of FGF23 with several cardiorenal pathophysiologic processes, as its relationship to at least some parameters such as sodium avidity and neurohormonal activation may be confounded by concomitant disease severity markers (some authors reported its related elevated level as a mere surrogate marker) [29]. Our findings in aggregate can further substantiate FGF23 as a potential diagnostic marker, particularly in the presence of combined cardiac and renal dysfunction and even its role as a causal intermediary deserves investigation.

Certain clinical investigations indicate that SuPAR has significant discriminative value for kidney disease; nonetheless, evidence-based validation remains insufficient [30]. Our research has shown that smoking correlates with significantly elevated SuPAR levels in AHF. This is consistent with another research [31]. These distinctions are unknown; however, hypertension, diabetes, and chronic renal disease are risk factors independently linked with greater SuPAR levels that may partly explain their effects [31]. This study supports our findings that AHF patients with hypertension or diabetes had significantly higher SuPAR levels. Patients without comorbidities may have increased SuPAR levels due to persistent inflammation and undiscovered illnesses such as rheumatologic or inflammatory bowel disorders. In-depth characterization of those with high SuPAR levels but no CV risk factors will illuminate the issue.

SuPAR (soluble urokinase plasminogen activator receptor) also showed a substantial diagnostic performance, with AUCs ≥ 0.88 for all main subgroup comparisons and high discrimination between AHF with or without renal dysfunction (AUC = 0.988). This is consistent with abundant evidence to suggest that SuPAR is an inflammatory biomarker indicative of immune activation and chronic inflammatory load, which forms the basis for both cardiovascular and renal disease pathways [32]. Meta-analyses confirm it could be used as an exploratory diagnostic marker of kidney disease, with a comprehensive sensitivity of 0.77 and a comprehensive specificity of 0.64 [33]. Increased SuPAR is linked to heart failure occurrence, development, and all-cause mortality, as well as risk of cardiovascular events independent from traditional risk markers [34].

Our finding showed that in AHF, hypertensive individuals had considerably higher NAG gene expression than those without. This is consistent with previous studies that found that increased diastolic pressure correlated with higher serum NAG levels [35,36]. NAG gene expression was related to a series of inflammatory markers, acid–base homeostasis, and renal function, not only in AHF but also in renal disease, supporting its implication as an early marker of tubular damage all across the cardiorenal continuum. The strong association of NAG with WBC count in AHF patients points to CRS-induced tubular stress [37]. Renal reactions to respiratory and metabolic challenges appear associated with pH, pCO₂, and bicarbonate, whereas the negative regression slope for oxygen tension reflects renal hypoxia in acute-on-chronic decompensation tubular damage [38]. There is sensitivity in the NAG for tubular failure and multisystemic involvement in renal sickness because it correlates well with creatinine, inflammatory indices, platelets, hepatic enzymes, and electrolytes.

NAG had the highest AUC for distinguishing AHF and AHF with renal dysfunction from controls (AUC = 1.0). Early response to tubular and proximal nephron stress from metabolic and hemodynamic derangements in HF and CR diseases could play a role in defining these patient populations. The highest levels of NAG indicate more severe heart failure and a worse prognosis (since this enzyme as a marker of renal tubule damage in urine or serum has been linked to structural and functional impairment) [39,40].

NAG was sensitive in differentiating AHF with renal insufficiency from primary kidney disease, indicating that while the sensitivity of NAG for the presence of renal disease in systemic conditions could be high, its specificity could fall under similar clinical circumstances. This further proves that proximal tubular markers such as NAG, reflective of early injury, may not differentiate multifactorial etiologies in all cases [37].

In our study the diagnostic accuracy of NAG as a mediator to differentiate between AHF with kidney disease and isolated renal diseases was poor, which might be due to the fact that it is highly sensitive for detecting renal involvement in systemic diseases, but its specificity declined in overlapping clinical entities. This finding is in line with previous reports that proximal tubular biomarkers, including NAG, sensitive to early injury, do not distinguish among etiologies of kidney disease when it is multifactorial [41]. Based on the current evidence and reports, it seems possible that these markers could provide extra ways to assess the cardiorenal effects when patients are admitted, particularly in intensive care units and cardiology departments that need quick clinical evaluations. FGF-23 and SuPAR seem to be promising biomarkers for renal and inflammatory activation, whereas NAG indicates tubular damage. Their use in combination with classical laboratory indices, natriuretic peptides, and clinical judgements could increase sensitivity for the early detection of complex patterns of cardiorenal overlap. Nevertheless, generalized use in day-to-day general internal medicine practice would need further confirmation in larger multicenter studies.

There are important limitations to the present study that should be addressed. First, it was a single-center pilot study with a small sample size that may not be generalizable and exhibits the potential to report false positive results—especially for ROC curve analyses claiming high discriminatory performance. The second limitation is that, because the study looked at data from a single point in time, we could compare how well different groups were diagnosed when they were admitted, but we couldn’t see how these results might change over time or if one factor caused another. Third, despite consideration of multiple biomarkers, no explicit composite multimarker model was developed, and the differential diagnostic gain compared with conventional markers such as serum creatinine and natriuretic peptides was not formally quantified. Fourth, heterogeneity even among patients with kidney disease (other than variation in chronicity and severity) could have included group effects upon biomarker expression. Last, lack of external validation in an independent cohort restricts verification of robustness. Large, multicentric prospective studies with longitudinal follow-up and formal multimarker modelling are needed to validate and extend the current findings.

The main clinical implication of the present study is that phenotypic characterization of cardiorenal may be improved with molecular and protein markers at presentation. Instead of serving as substitutes for traditional markers, they could also be used to supplement clinical assessment and standard biochemistry to provide a higher diagnostic accuracy in complicated presentations of AHF with renal dysfunction.

## Conclusions

FGF23, SuPAR, and NAG could serve as potential markers of AHF-induced cardiorenal dysfunction. FGF23 and SuPAR might be predictive of renal involvement and complex cardiorenal diseases. Inflammation, acid–base status, hypoxia, and renal function shape NAG gene expression as a marker of early renal damage before serum creatinine. To optimize discrimination, diagnostic accuracy, and clinical decision-making for patients with AHF and concomitant renal disease, we propose that novel biomarkers should be based on defining the molecular characteristics of this condition.

## Data Availability

Data is provided within the manuscript.

## Abbreviations

AHF: Acute heart failure
FGF23: Fibroblast growth factor-23
SuPAR: Soluble urokinase plasminogen activator receptor
NAG: N-acetyl-β-D-glucosaminidase
CVDs: Cardiovascular diseases
BNP: B-type natriuretic peptide
